# Hunting for vital nodes in complex networks using local information

**DOI:** 10.1038/s41598-021-88692-9

**Published:** 2021-04-28

**Authors:** Zhihao Dong, Yuanzhu Chen, Terrence S. Tricco, Cheng Li, Ting Hu

**Affiliations:** 1 Department of Computer Science, Memorial University of Newfoundland, St. John’s, A1C 5S7 Canada; 2 Verafin Inc., Data Scientist, St. John’s, A1A 0L9 Canada; 3 Department of Electrical and Computer Engineering, Memorial University of Newfoundland, St. John’s, A1C 5S7 Canada; 4 School of Computing, Queen’s University, Kingston, K7L 3N6 Canada

**Keywords:** Computer science, Computational science, Epidemiology

## Abstract

Complex networks in the real world are often with heterogeneous degree distributions. The structure and function of nodes can vary significantly, with vital nodes playing a crucial role in information spread and other spreading phenomena. Identifying and taking action on vital nodes enables change to the network’s structure and function more efficiently. Previous work either redefines metrics used to measure the nodes’ importance or focuses on developing algorithms to efficiently find vital nodes. These approaches typically rely on global knowledge of the network and assume that the structure of the network does not change over time, both of which are difficult to achieve in the real world. In this paper, we propose a localized strategy that can find vital nodes without global knowledge of the network. Our joint nomination (JN) strategy selects a random set of nodes along with a set of nodes connected to those nodes, and together they nominate the vital node set. Experiments are conducted on 12 network datasets that include synthetic and real-world networks, and undirected and directed networks. Results show that average degree of the identified node set is about 3–8 times higher than that of the full node set, and higher-degree nodes take larger proportions in the degree distribution of the identified vital node set. Removal of vital nodes increases the average shortest path length by 20–70% over the original network, or about 8–15% longer than the other decentralized strategies. Immunization based on JN is more efficient than other strategies, consuming around 12–40% less immunization resources to raise the epidemic threshold to $\tau \sim 0.1$. Susceptible-infected-recovered simulations on networks with 30% vital nodes removed using JN delays the arrival time of infection peak significantly and reduce the total infection scale to 15%. The proposed strategy can effectively identify vital nodes using only local information and is feasible to implement in the real world to cope with time-critical scenarios such as the sudden outbreak of COVID-19.

## Introduction

In the real world, we are surrounded by complex systems, such as social cooperation systems^1^, communication infrastructures^2^, and transportation systems^3^. Complex networks can represent these systems and help researchers to understand them deeply^4^. Spreading phenomena in networks are crucial in studies of complex networks, since they can be used in many aspects of life, such as stopping contagious disease^5^ and improving information diffusion^6^. These two examples show opposite directions where the spreading process can be affected. Removing a fraction of structural nodes, i.e., vital nodes, would break a network and prevent the spread of a large scale epidemic. In contrast, diffusing information from a set of origin nodes, i.e., influential nodes, would maximize the influence scale in a network. Previous research has pointed out that vital nodes and influential nodes are not equivalent in a network^7,8^. Many approaches to identifying influential nodes have been proposed to promote the spreading process in networks^9–19^. On the other hand, vital nodes also have a profound effect on network spreading phenomena in complex networks because the integrity and stability of complex networks hinge on them^20–22^. Identifying these critical nodes is also crucial in controlling the spreading process for information, behaviors, pathogens, and more^9,14–19,23,24^. For example, removing or vaccinating the vital nodes will impede the spread of contagious disease. Monitoring the vital nodes’ abnormal behavior in the financial transaction network can help the financial institution detect frauds efficiently and effectively.

In recent years, many researchers have studied how to identify the vital nodes in complex networks. Some of them propose new centrality measures and provide more metrics for measuring node importance, such as PageRank^25^, VoteRank^14^, WVoteRank^26^, LocalRank^11^, ClusterRank^12^, Coreness^9^, LeaderRank^27^, and TwitterRank^28^. These proposed centralities consider more information than the traditional ones such as degree, closeness^29^, betweenness^30^ centralities, but require more computational time. Others develop new algorithms to search for the prominent nodes in the network. SPIN approach^10^ runs faster than greedy algorithms with little quality loss, but is still not efficient enough to be applied to large scale networks^14^. Some fast heuristic algorithms are proposed to cope with this problem. Chen et al. develop a degree discount algorithm^31^, which is more than one million times faster than a typical greedy algorithm^32^, while holding a similar accuracy performance to the greedy algorithm. For the networks with community structures, He et al. propose a community-based method^33^ to search for vital nodes from different communities with the community detection algorithms^34^. Morone and Makse map the vital nodes identification problem onto optimal percolation in random networks to find the minimal set of critical nodes^35^. However, most of the previous algorithms are centralized and need full knowledge of the network structure. It is often challenging to acquire the complete information of the network in practice because of the scale and dynamics of the network and privacy concerns. Moreover, the previous strategies mainly focus on identifying vital nodes in undirected networks, while only few of them are able to work on directed networks, a limitation that cannot be ignored in practice.

To cope with these challenges, we propose a localized and decentralized approach to hunt for vital nodes without the network’s global information, called the Joint Nomination (JN) strategy. This strategy randomly selects a fraction of nodes in the network as nominators and lets each nominator nominate one of its neighbours, called co-nominator, and then selects a node from the common neighbours of the nominator and co-nominator. The nodes selected from common neighbours are the final identified vital nodes.

The proposed JN strategy has several advantages compared to the existing methods. First, it is decentralized and does not need to access the whole network. Second, its computation time is independent of the network scale, only depending on the number of vital nodes required to be found and on the network density. Third, this proposed method is feasible to implement in the real world and capable of being applied in a time-critical scenario such as the sudden outbreak of COVID-19. In contrast, most of the existing methods involve iterative global search, and they are inefficient and can be impractical in many cases. The proposed JN strategy is also a general approach to finding vital nodes in both undirected and directed networks, and has many applications. For example, finding the sink hubs (high in-degree) and source hubs (high out-degree) in a transaction network is important in detecting financial frauds and money laundering for financial institutions. Identifying and immunizing a set of vital nodes in a contact network can help to impede the spread of contagious disease, such as COVID-19.

The performance of our strategy is tested based on the two application scenarios above. The transaction network is a directed network. The average degrees of the identified node sets are measured to evaluate the approach’s ability to find hubs. The contact network where disease spread is undirected, and we evaluate the pathogen spreading features on the remaining networks after removal of vital nodes found by our proposed strategy.

## Strategies

First, we present our joint nomination (JN) strategy, and the baseline strategies that we compare against, specifically the Site Percolation (SP) and friend nomination (FN) strategies.

**Figure 1:**
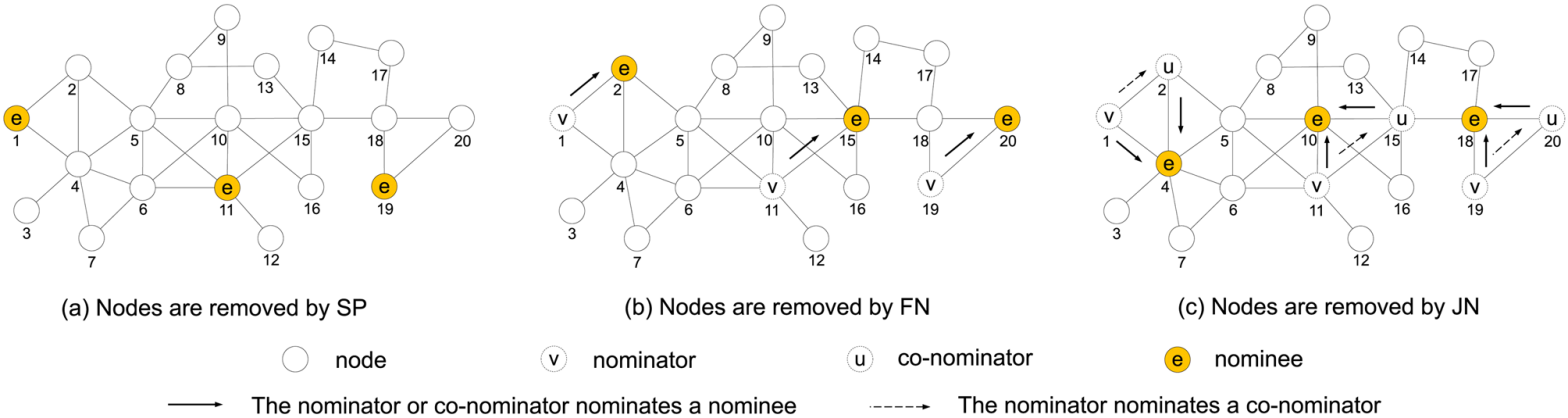
Illustration of three decentralized vital node identification strategies.

### Site percolation (SP) strategy

The SP strategy randomly selects nodes from the network. This strategy does not require global knowledge of the network in the selection of any individual node. It is trivial to implement, but does not offer any advantage in identifying vital nodes. The SP strategy is included to show the minimum performance threshold of any other strategy.

Figure 1a shows the process of the SP strategy, which chooses a fraction of nodes from a network randomly. In this example, nodes 1, 11 and 19 are chosen by the strategy.

### Friend nomination (FN) strategy

The Friendship Paradox (FP) ^36^ states that a person’s friends are more popular than himself or herself on average. In other words, the average degree of a node’s neighbours is greater than that of itself. The Friend Nomination (FN) strategy is based on the FP and is an efficient way to find nodes with relatively high degrees. It is used in acquaintance immunization ^37^.

The FN strategy is localized and easily implemented in real cases. First, it randomly picks a fraction, *f*, of nodes in the network, which are called *nominators*. Second, each node in the nominator set randomly nominates a node from its neighbours, generating a new set of nodes, *nominees*. The set of nominees are the chosen vital set of nodes, and have the same size as the set of nominators.

The probability of the nominator with degree *j* connecting with the nominee with degree *k* is $kj/(2E-1)$. Assuming the nominator is connected with the nominee, then the probability that this nominee is nominated is 1/*j*. For a specific node with degree *k*, if any node except itself in the network is selected in the first step, there is an opportunity that this node with degree *k* can be nominated. The probability that any node is picked uniformly at random from the network is 1/*N*, then

1 $$\begin{aligned} \begin{aligned} p(k)&= \sum _{N-1}\frac{1}{N}\left( \frac{kj}{2E-1}\times \frac{1}{j}\right) \\&=\frac{N-1}{N}\times \frac{k}{2E-1}. \end{aligned} \end{aligned}$$

This shows that the probability of a node becoming nominated with the FN strategy is determined by its degree, *k*. This explains why the FN strategy will preferentially select higher-degree nodes of the network.

Figure 1b illustrates the FN strategy. FN uses the randomly selected nodes 1, 11, and 19 as the nominator set. Each of these nodes nominates one of its neighbours randomly as nominees, choosing nodes 2, 15 and 20. These nominees are selected as the vital nodes in the network.

### Joint nomination (JN) strategy

Inspired by FN, we develop a new decentralized vital node identification strategy: the Joint Nomination (JN). We illustrate the operating principle of the JN strategy on undirected networks. It can be applied to directed networks with little modification. First, we randomly select a fraction, *f*, of nodes, which are called nominators. For each nominator, a *co-nominator* node is chosen. The co-nominator is randomly selected from the neighbour nodes of the nominator, called the co-nominator candidates. One node is randomly chosen as a nominee from the common neighbours of the nominator and its co-nominator. If the nominator and its co-nominator do not have a common neighbour, another co-nominator is chosen from the co-nominator candidates, continuing until a nominee is selected. If no nominee can be found, we randomly select a co-nominator candidate as a nominee.

An example of the JN strategy for undirected networks is shown in Fig. 1c, where nodes 1, 11, and 19 are randomly selected as nominators. Next, each selected node nominates a random neighbour as a co-nominator. In this example, nodes 2, 15, and 20 are co-nominators. Finally, each pair of nominator and co-nominator chooses a nominee from their common neighbours. In this figure, nodes 4, 10, and 18 will be nominated as the vital nodes. All of these steps are localized, decentralized, random, and do not require full knowledge of the network.

Based on this idea, we develop three variants: identifying hubs in undirected networks, identifying source hubs in directed networks, and identifying sink hubs in directed networks. There are two kinds of neighbours for any node in a directed network: predecessors and successors. A node’s predecessor refers to its a neighbour node that has a link pointing to it, and a node’s successor is a neighbour to which it points. There are also two kinds of hubs in directed networks: source hubs and sink hubs. Source hubs refer to nodes with high out-degree, while sink hubs represent those with high in-degree. The predecessors act as neighbours for our JN strategy when the target nodes are source hubs, while the successors are regarded as neighbours when the target nodes are sink hubs. The algorithm of the proposed JN strategy in undirected and directed networks is summarized in Algorithm 1.

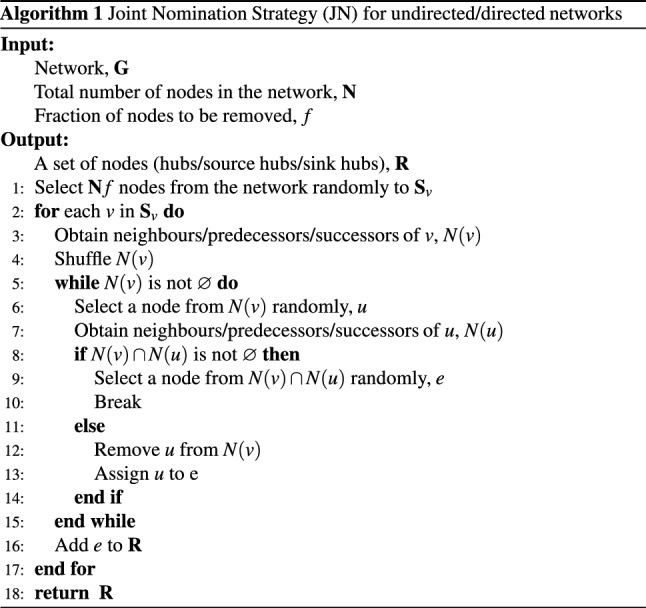



**Figure 2:**
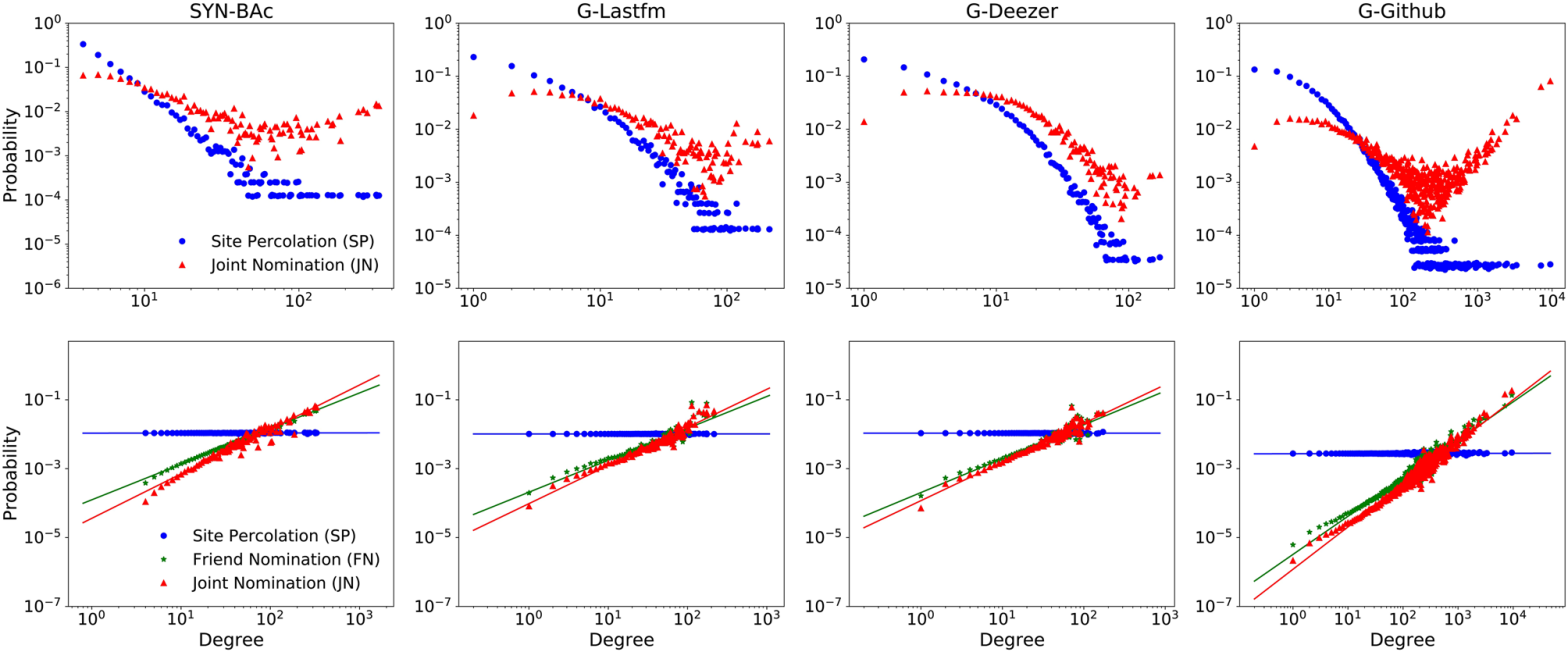
Degree distributions of nodes sampled with the SP and JN strategies (top row), and the degree distributions of the selected nodes normalized by the original network distribution (bottom row). The JN strategy shows a preference for selecting higher-degree nodes, and has a distribution skewed more towards higher-degree nodes than the distributions obtained with the FN strategy.

**Table 1:** Slopes of the normalized degree distributions for different networks and strategies.

Network	Strategy	Slope	Slope variance
SYN-BAc	SP	0.0007	3.6771e−06
SYN-BAc	FN	1.0700	1.7241e−04
SYN-BAc	**JN**	**1.3487**	1.1991e−03
G-Lastfm	SP	− 0.0019	2.6167e−06
G-Lastfm	FN	0.9262	1.1843e−03
G-Lastfm	**JN**	**1.1068**	9.6201e−04
G-Deezer	SP	− 0.0057	1.1438e−05
G-Deezer	FN	0.9818	1.4423e−03
G-Deezer	**JN**	**1.1224**	1.0440e−03
G-Github	SP	0.0005	3.2787e−06
G-Github	FN	1.1083	1.3221e−04
G-Github	**JN**	**1.2306**	1.8351e−04

## Results

In this section, we experimentally validate the effectiveness of the JN strategy on the task of finding hubs in both undirected and directed networks. Identifying and immunizing hubs in a social contact network will impede the disease spread significantly, because this network features with a long-tail degree distribution in most cases. We also conduct a network spreading simulation on the original networks and the remaining networks after removing nodes identified by the proposed JN strategy. We use the SIR model in this simulation. The experiments are performed on an Ubuntu 18.04.3 LTS system with Lenovo ThinkStation, Xeon 24 cores, 64 GB RAM, and a clock speed of 3.2 GHz.

### Degree distribution of selected vital nodes

An efficient high-degree nodes identification strategy should show a preference for high-degree nodes. To test the ability of the various strategies to select high-degree nodes, a node is chosen randomly, then the selection strategy is used to identify the chosen node. This procedure is repeated ten million times to build the degree distribution of selected nodes for each strategy. The SP, FN and JN strategies are used on four undirected networks. The degree distribution of the samples reflect each strategy’s capability to find hubs in a network.

Figure 2 shows the obtained degree distributions for each network and strategy. The four plots in the top row are the observed degree distributions of the selected vital nodes for each network. The distributions in the bottom row are obtained by dividing the distribution of the vital set by the network’s degree distribution, which avoids the influence of the original degree distribution.

The degree distribution of nodes sampled with the SP strategy is very close to the network’s degree distribution, evidenced by the horizontal normalized degree distribution in the bottom panel. This strategy shows no preference for nodes of any degree. The JN strategy improves the probability of selecting the high-degree nodes, with a lower probability of selecting low-degree nodes. The normalized degree distributions for the JN strategy have positive slopes, implying that the JN strategy tends to identify nodes with high degrees. Table 1 lists the slopes of the normalized degree distributions for all strategies and networks. The slopes of the normalized degree distributions for the nodes selected using the JN strategy are significantly higher than that obtained with the FN strategy, e.g., 1.3487 for JN compared to 1.07 for FN on SYN-BAc network.

**Figure 3:**
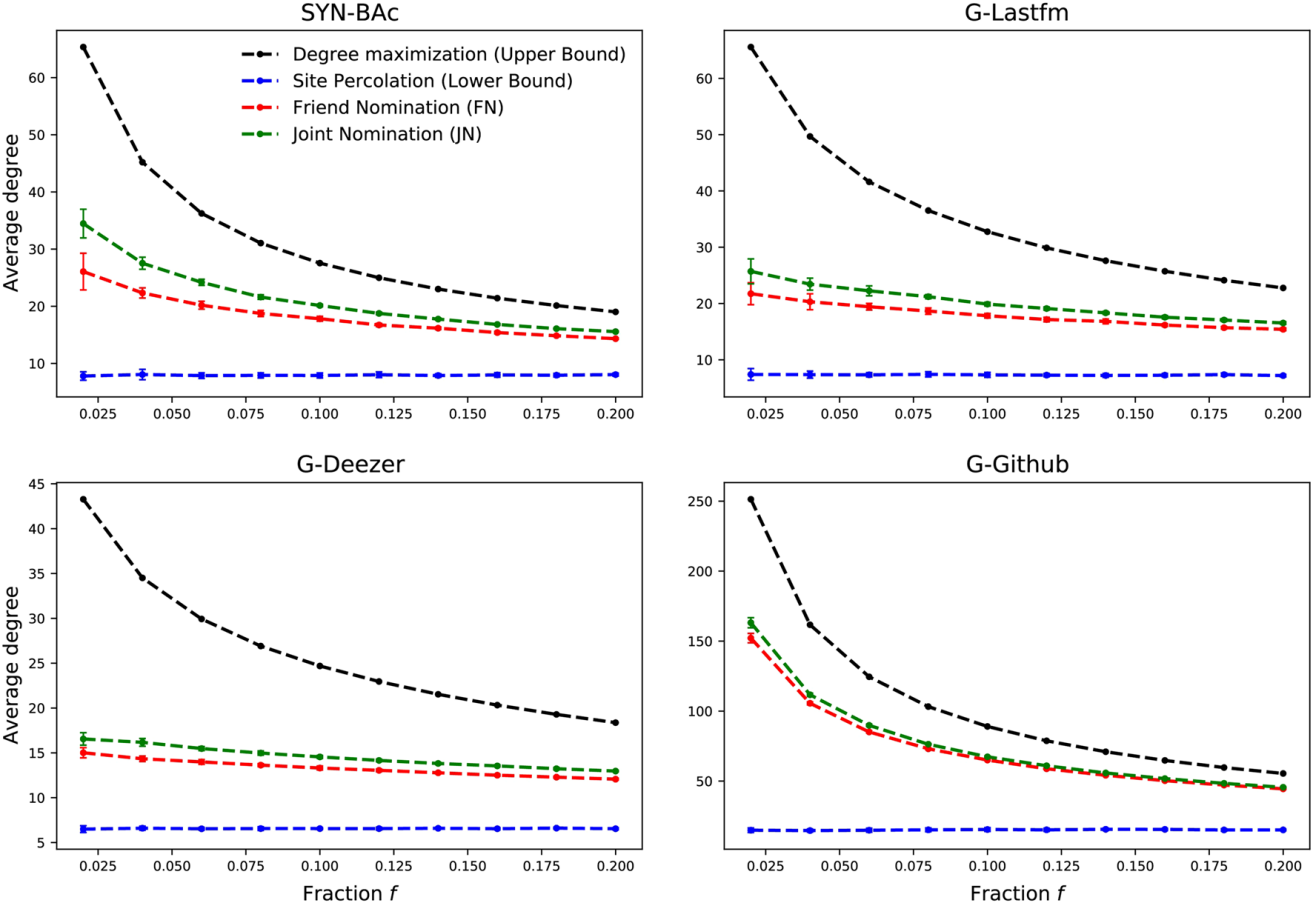
The average degree of the identified node set by different approaches on four datasets. The average degree of nodes identified with JN is always greater than that with FN or SP in each fraction and each dataset. With the increasing of the fraction, the gap between JN and FN shrinks, because there are not enough high degree nodes left in the network. This implies that JN can find high degree nodes more efficiently than FN. The results are averaged over 50 independent runs.

**Figure 4:**
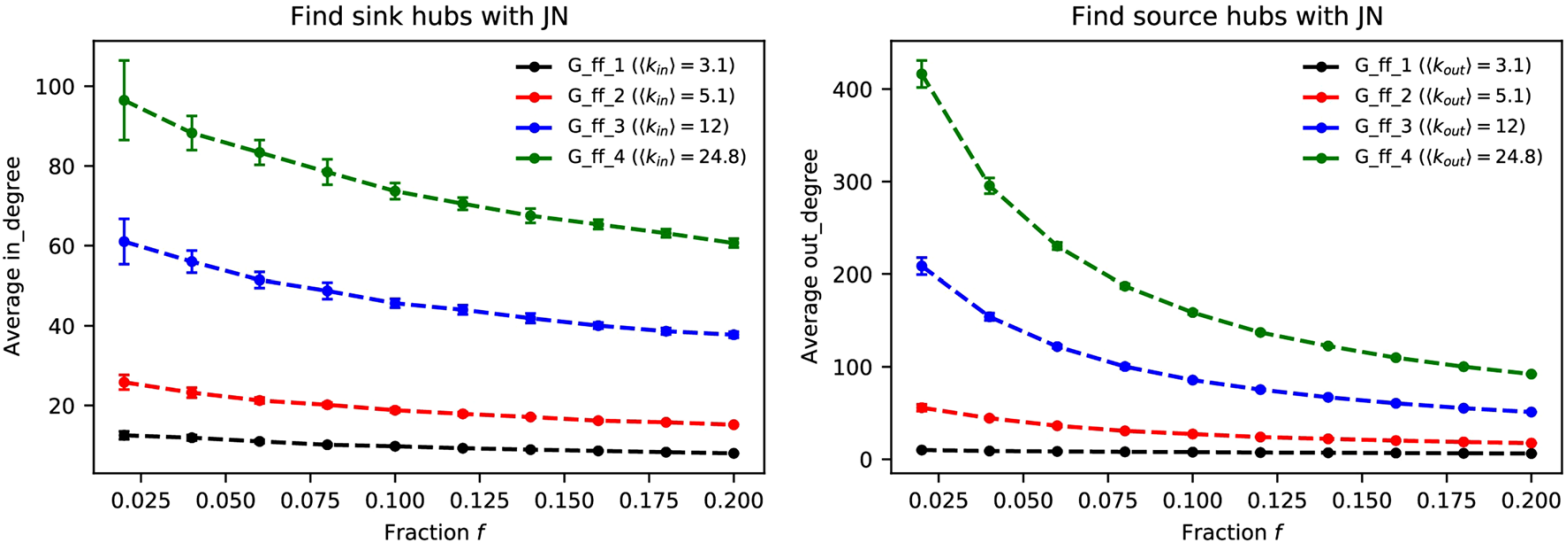
How the network’s average degree affects JN’s performance on finding hubs in directed networks. All of he four generated networks have the 8000 nodes, the average degrees are 3.1, 5.1, 12, 24.8, respectively. JN performs better in the network with higher average degree. The results are averaged over 50 independent runs.

**Figure 5:**
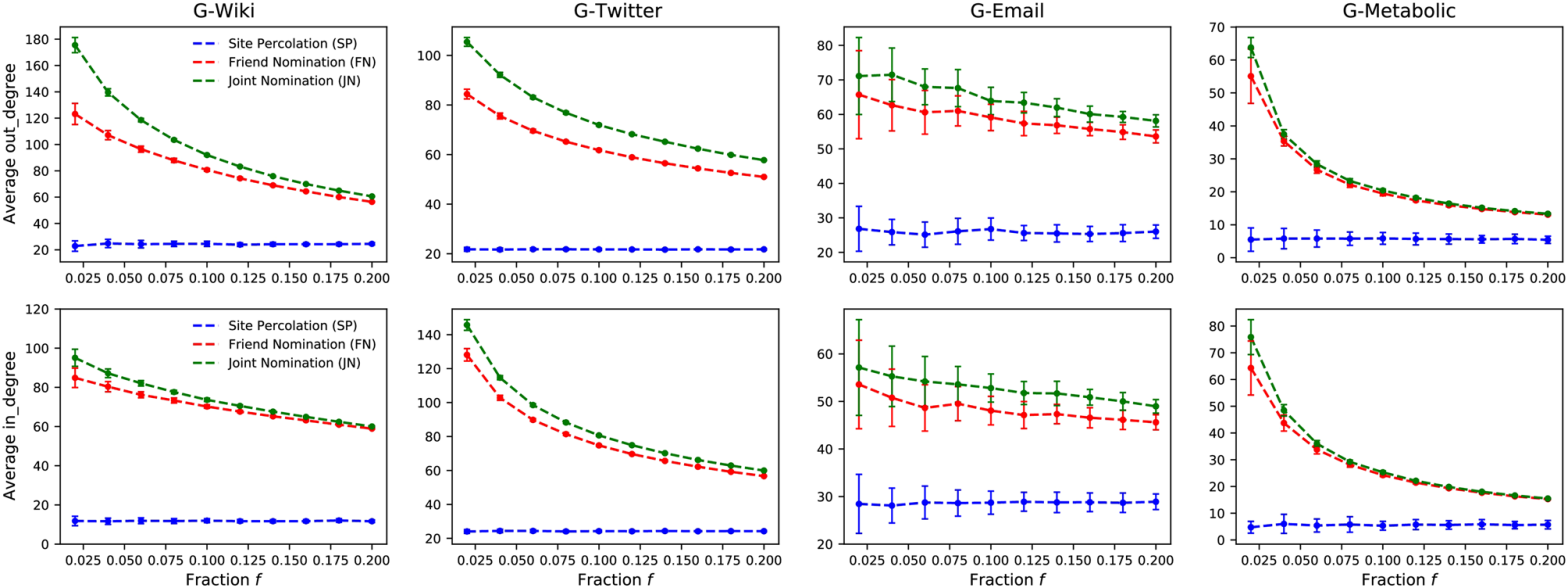
The average in-degree and out-degree of the selected vital nodes using the SP, FN and JN strategies (blue, red and green lines, respectively) on four real-world directed networks. The results are averaged over 50 independent runs.

### Average degree of selected nodes

In this section, we use the different selection strategies (SP, FN, and JN) on both undirected and directed networks to select sets of nodes ranging from 2–20% of the original network size ($f=0.02$–0.2). The average degree of the identified set of nodes is measured for each method. The average degree metric reflects the performance of each strategy on finding high-degree nodes. The simulations are repeated for 20 times.

Figure 3 shows the arithmetic mean of the identified nodes’ degrees on four undirected networks (SYN-BAc, G-Lastfm, G-Deezer, and G-Github). The uncertainty represents the standard deviation of the averaging. For smaller fractions, the average degrees of the identified nodes are relatively large for the two nomination strategies, with the JN strategy providing further improvement over the FN strategy. The standard deviation is also largest for smaller fractions, but the average degree of the nodes selected with the JN strategy is still substantially higher than that from the FN strategy. As the fraction of identified nodes increases, the average degree of the identified set decreases. This is mainly because the number of hubs is limited in these networks. With the SP strategy, the selected nodes’ average degrees stay effectively constant as the fraction of selected nodes is increased, remaining roughly equal to the average degree of the whole network. Thus, the SP strategy represents the worst-case performance of any vital node identification strategy. Best-case performance is shown by selecting only the highest-degree nodes (black line), representing strategies that require global knowledge of the network. Both the FN and JN strategies are effective at finding hubs, but the JN strategy always outperforms the FN strategy, especially when the selected fraction is small.

Figure 4 shows the results for four synthetic directed networks with different average in-degrees and out-degrees generated by the Forest Fire model. It can be seen that the average in-degree and average out-degree of the original networks can affect the average degree of the nodes identified by the JN strategy. When the network is sparse (low average degree), the diversity of nodes’ degrees is low. In other words, the network is homogeneous. For these networks, the out-degree nodes are more heterogeneous than the in-degree nodes, with higher degree on average than the in-degree nodes. In this case ($k_{in}$ = 3.1 and $k_{out}$ = 3.1), the JN strategy can find high-degree nodes, but we can hardly observe it very clearly, such as the black dashed lines with $k_{in}$ = 3.1 and $k_{out}$ = 3.1. However, for a denser networks, we can see that an obvious improvement on both average in-degree and average out-degree, such as green dashed lines with $k_{in}$ = 24.8 and $k_{out}$ = 24.8. This implies that when there are some obvious hubs in the networks, JN can find them efficiently.

Figure 5 shows the results of simulations on real-world directed networks, including social networks and biological networks. The top row shows the average in-degree and the bottom row the average out-degree. In all cases, the proposed JN strategy outperforms the FN strategy, except for the G-Metabolic network, where the performance gain is slight.

### Average shortest path length of the remaining network

Removing the vital node set identified with the hub-finding strategy can alter the structure of the remaining network. If the average shortest path length of the remaining network is longer, then information or disease spread becomes slower, taking more time to reach the full network. Calculating the average shortest path length of the remaining network on a large-scale network is time consuming, i.e., $\mathcal {O}$($n^3$). The average shortest path length in our remaining networks is calculated by randomly selecting 5% of the nodes as reference nodes, and calculate the average shortest path length between each reference node and all other nodes. To reduce the uncertainty, the mean of 50 independent runs is used.

**Figure 6:**
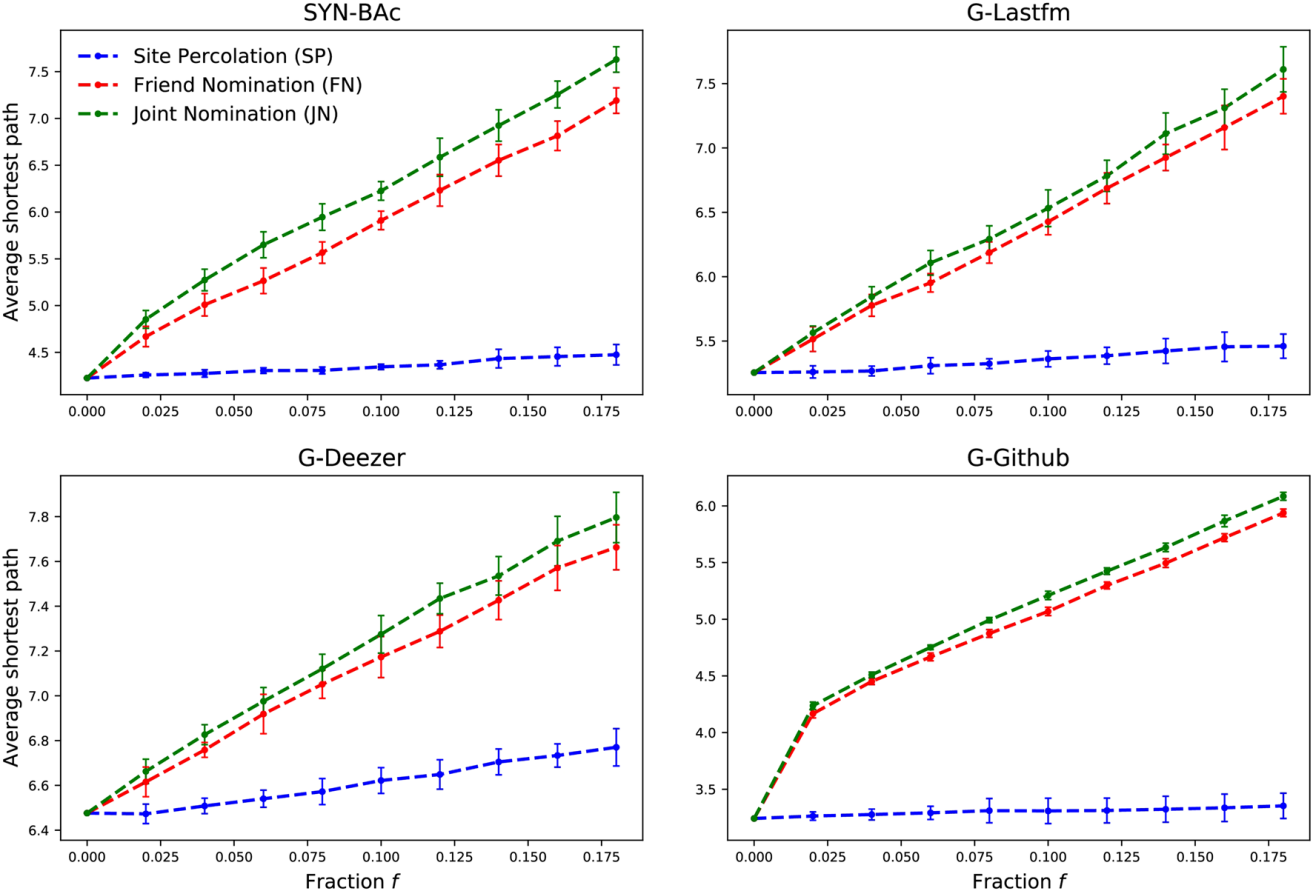
The average shortest path length of the remaining network after nodes removal with SP, FN and JN. The results are averaged over 50 independent runs.

Figure 6 shows the average shortest path length of the remaining networks after removing an increasing fraction of nodes ($f=0.02$–0.18) identified by different strategies. There is a clear rise of the average shortest path length as the fraction of nodes removed increases, with the JN strategy always performing better than the FN strategy for all networks tested. This means that node removal with the JN strategy can increase the average shortest path length more efficiently than the FN strategy, reducing the pathogen or information propagation rate in the network.

**Figure 7:**
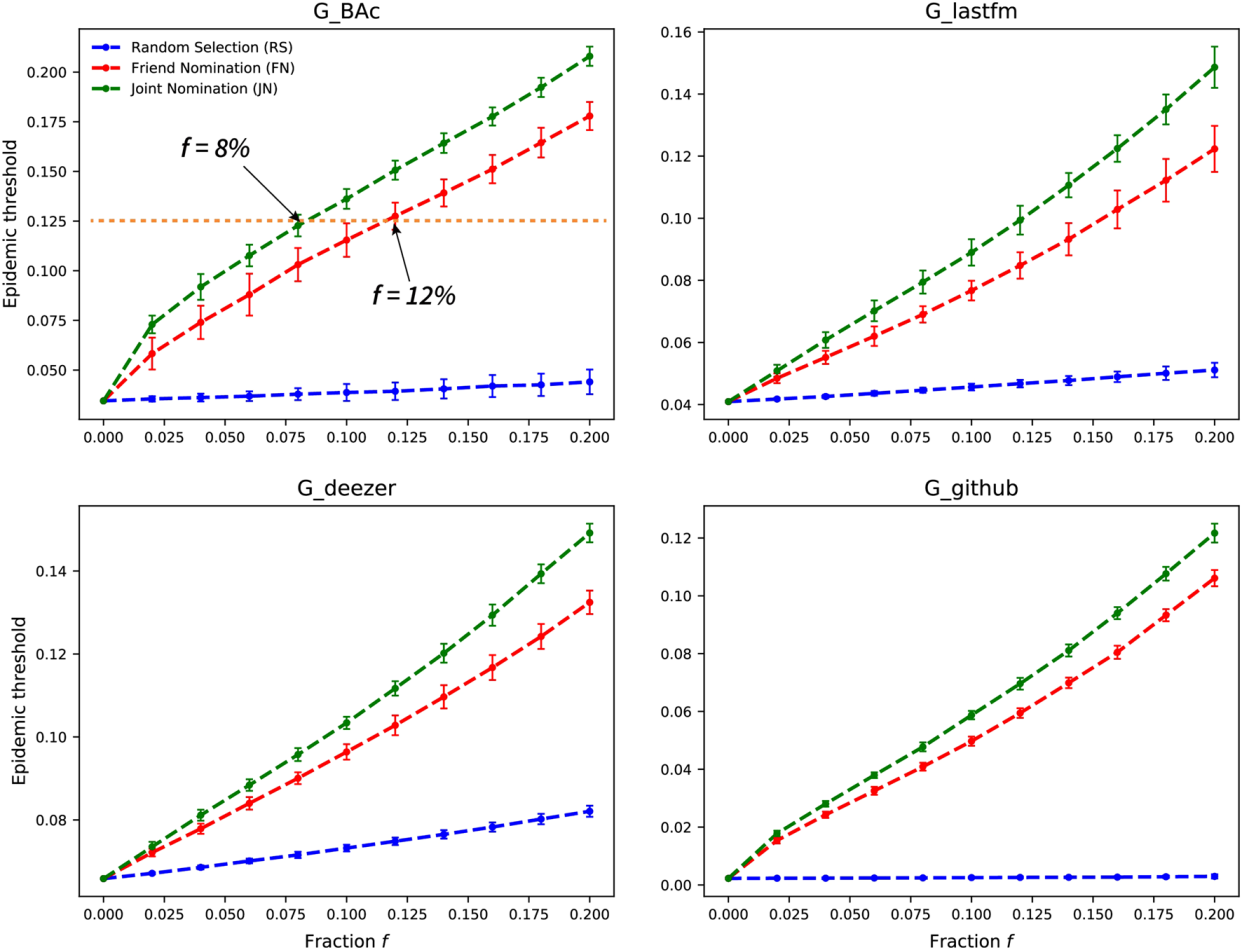
The trend of epidemic threshold with removing nodes identified with different strategies. Epidemic threshold for SP (blue, dashed line), FN (red, dashed line) and JN (green, dashed line) for SYN-BAc, G-Lastfm, G-Deezer and G-Github networks. The nomination strategies outperform the site percolation strategy with JN providing further improvement over FN. The results are averaged over 50 independent runs.

### Epidemic threshold of the remaining network

The impact of removing hubs using the hub-finding strategies is measured by calculating the epidemic threshold, $\tau$, (Eq. (3)) of the remaining network. The epidemic threshold is a crucial indicator reflecting a network’s capability of resisting the spreading of pathogens. A pathogen can prevail only if its spreading rate exceeds the epidemic threshold of the network^4^. Thus, the greater epidemic threshold a network has, the harder the network is for pathogens to spread. In this experiment, the mean epidemic threshold, $\langle \tau \rangle$, for each strategy is calculated using the average of epidemic thresholds from the results of 50 simulations.

Figure 7 shows the efficiency of different strategies on increasing epidemic threshold. The proposed JN strategy always needs the least node removal to achieve a certain epidemic threshold, meaning that it has the highest immunization efficiency. For example, to increase the mean epidemic threshold from $\langle \tau \rangle =0.033$ to 0.125 on SYN-BAc network, 12% of nodes identified by the FN strategy have to be immunized, compared to only 8% of nodes with the JN strategy, representing a 33.3% efficiency gain. On G-Github network, the initial epidemic threshold is nearly zero, which means that G-Github network is highly conductive, such that any disease, even with a very low spreading rate, can lead to an epidemic. For 20% node removal, the JN strategy increases the epidemic threshold to $\langle \tau \rangle \sim 0.12$, while the FN strategy raises it to $\langle \tau \rangle \sim 0.1$. The SP strategy hardly increases the epidemic threshold, which means it only protects the immunized individuals without much influence on the network structure property. The two nomination strategies can not only protect the immunized individuals, but also bring an extra benefit of making the network more difficult for disease to spread. The proposed JN strategy performs better than the FN strategy in raising epidemic threshold for all four network datasets.

### SIR simulation results

In this section, network spreading simulations using the SIR model are conducted on the four undirected networks of SYN-BAc, G-Lastfm, G-Deezer, G-Github. First, we compare the two localized strategies, i.e., the proposed JN and the FN, on the task of mitigating the spreading rate and infection scale on networks. Since most of the recent work about influential nodes identification, such as *k*-shell decomposition^9^ and VoteRank^14^, are global and rely on the full information of the network, we also compare the localized JN with them to investigate the room for improvement. In the first experiment, a fraction $f = 0.1$, 0.2, and 0.3 of the nodes identified with the FN and JN strategies are removed from each original network. In the second experiment, a fraction $f = 0.1$ of the nodes identified with the JN (localized), *k*-shell (global) and VoteRank (global) strategies are removed from each original network. SIR simulations are performed on the reduced networks using the EoN (EpidemicsOnNetworks) model^38,39^. The simulations use $\beta =1.5$, $\gamma =1$, and $p=0.01$, where $\beta$ is the transmission rate per edge, $\gamma$ is the recovery rate per node and *p* is the ratio of the number of initially infected nodes to all of the nodes in the network.

**Figure 8:**
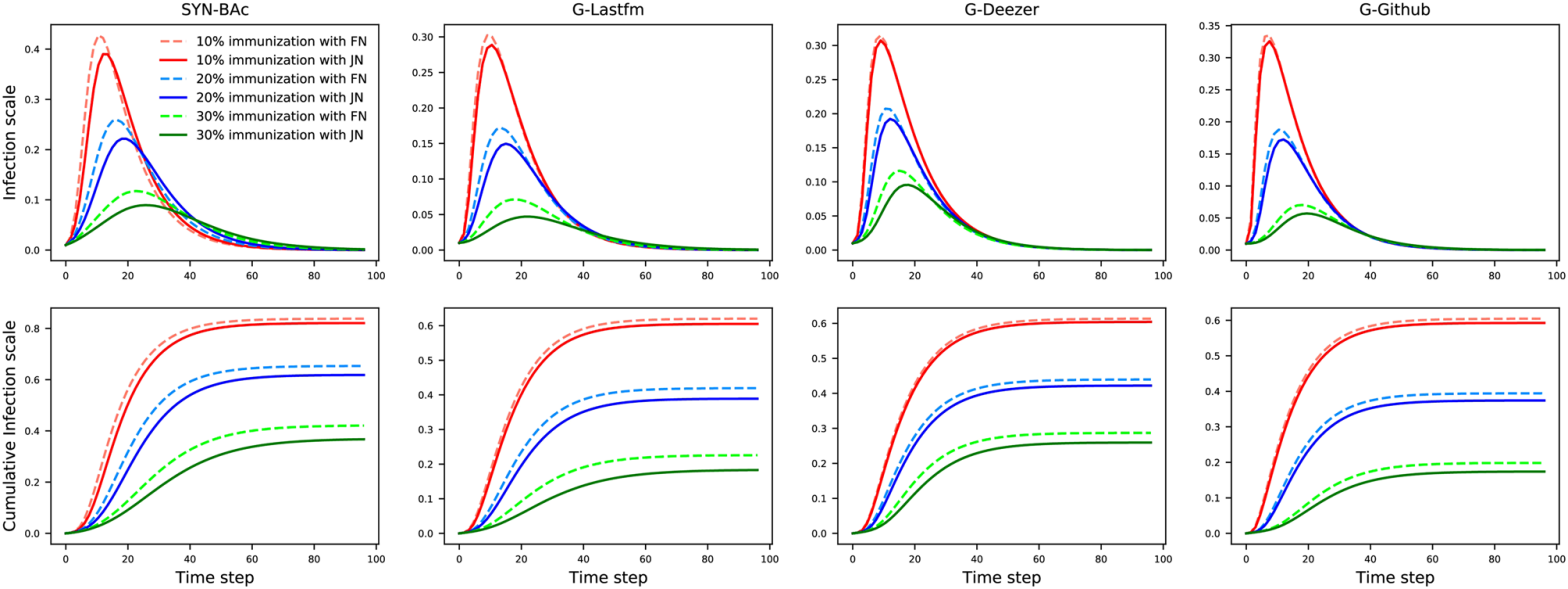
The infection scale (top row) and cumulative infection scale (bottom row) on four networks with different fractions of nodes removed under various strategies, where $\beta = 1.5$, $\gamma = 1$ and $p = 0.01$. In the top row, JN performs better than FN in all the fractions and all the datasets, since JN generates lower and postponed infection scale peaks compared to FN. With the increasing of the nodes removal fraction, the advantage of JN grows. In the bottom row, immunizing the same number of nodes identified by JN can reduce the network’s connectivity more than FN, resulting in a smaller final infection scale. For example, the final infection scale decreases from 40 to 32% when immunizing 30% nodes identified by JN compared with FN in SYN-BAc network. The results are averaged over 20 independent runs.

**Figure 9:**
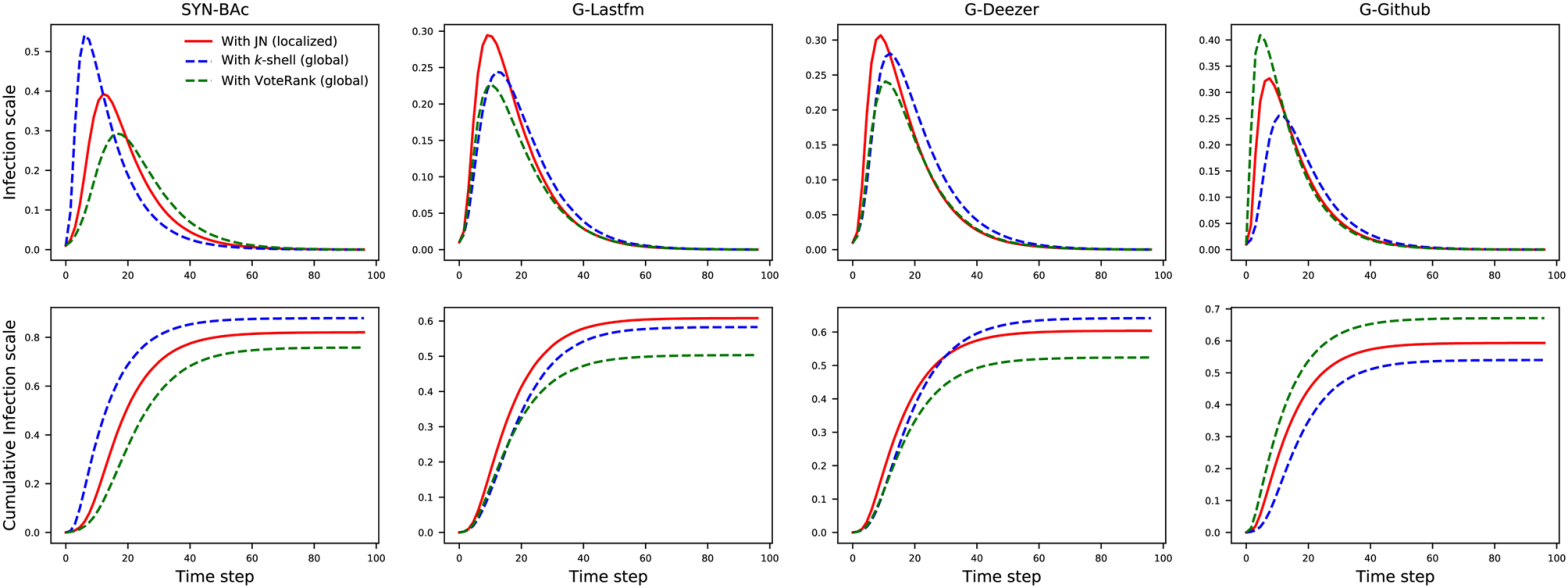
The infection scale (top row) and cumulative infection scale (bottom row) on four networks with 10% of nodes removed under various strategies, where $\beta = 1.5$, $\gamma = 1$ and $p = 0.01$. Immunizing the same number of nodes identified by JN, the top *k*-shell, and the top VoteRank leads to different changes to the network’s connectivity. JN reaches an intermediate final infection scale on SYN-BAc, G-Deezer, and G-Github networks. In the G-Lastfm network, JN shows the largest infection scale but is in fact only 0.04 greater than that of *k*-shell. The results are averaged over 20 independent runs.

Figure 8 shows the SIR simulation results of the JN and FN strategies. The top row in Fig. 8 shows the infection scale over simulation time. It can be seen that all the curves have the same trend. The infection scales have sharp increases at the beginning of the spreading process, reach a peak, then return down to almost zero. The reason for this shape is that there are many susceptible nodes at the beginning, and there is a large population of nodes that can be infected. As the population of susceptible nodes decreases, the recovery rate overtakes the infection rate, and the infection scale goes down. The differences between network, strategy and fraction of removed nodes are in the timing and height of the peaks. As more nodes are removed (larger *f*), the peak time is delayed and the peak height is lowered. This occurs because the network’s connectivity is decreased when more hubs are immunized. It is worth noting that, for any given *f*, nodes removed using the JN strategy can delay the infection scale peak’s arrival time and lower its height more than nodes removed using the FN strategy, for all cases. This implies that the JN strategy outperforms the FN strategy in the task of immunizing vital nodes to impede disease spread. The bottom row of Fig. 8 shows the cumulative infection scales’ rising time. We evaluate the cumulative infection scale, consisting of the infection scale plus the recovered scale. Thus, it rises monotonically. It is observed that the JN strategy always performs better than the FN strategy, since the final infection scale for the JN strategy simulations is smaller than that using the FN strategy in all networks.

Figure 9 shows simulation results of the JN, the top *k*-shell, and the top VoteRank strategies. In the SYN-BAc network, generated by the Barabasi-Albert preferential attachment model, since all the nodes have the same *k*-shell score, the top *k*-shell method degenerates to site percolation where nodes are removed randomly. Thus, the top *k*-shell method results in the largest final infection scale in the SYN-BAc network, despite being a global strategy. As expected, in the G-Lastfm network, the two global methods achieve a lower final infection scale than JN, but the gap between JN and the top *k*-shell is only within 0.04. Surprisingly, JN beats the top *k*-shell in the G-Deezer network, and the top VoteRank in the G-Github network, respectively. Therefore, this experiment shows that the performance of the proposed JN, only relying on local information, is competitive compared to the global methods.

## Discussion

In this paper, we have developed a decentralized strategy for identifying vital nodes in a network without requiring global knowledge of the network structure. The proposed JN strategy first randomly selects a set of nodes from the network as nominators. Each nominator obtains a co-nominator from its neighbours (predecessors or successors in directed networks). Then each pair of nominator and co-nominator nominates a nominee from their common neighbours. The nominees are the identified important nodes.

The effectiveness and efficiency of the proposed strategy were investigated by conducting experiments on both synthetic and real-world networks. These datasets include both undirected and directed networks. The degree distribution and average degree of nodes identified by the JN strategy showed that the JN strategy can find high-degree nodes in both undirected and directed networks. Compared to the FN strategy, higher-degree nodes took larger proportions in the degree distribution of the identified node set, and the average degree of the nodes was higher. Removing the nodes identified by the JN strategy raised the homogeneity of the remaining network. The average shortest path length and the epidemic threshold on the reduced networks were increased, and were larger than results obtained using the FN strategy. This implies that the proposed JN strategy can find vital nodes of the network efficiently.

SIR simulations were conducted on networks reduced by removing (immunizing) nodes identified by the localized JN and FN strategies, as well as the global *k*-shell and VoteRank methods. The network’s connectivity decreased dramatically for all strategies, delaying the arrival time of the peak infection scale, and reducing the peak height. The cumulative infection scale was also reduced. However, compared to the localized FN, our JN strategy was more effective at reducing the spreading speed, resulting in further delays to peak arrival, lowering peak height, and resulting in a smaller cumulative infection scale. This implies that the nodes identified by the JN strategy were crucial nodes in the network to impede disease flow. Compared to the global *k*-shell and VoteRank methods, the localized JN is still competitive in decreasing disease spread.

The computational complexity of the JN algorithm is low. The total computational time includes two nested loops. For the outer loop, the time to search for a nominee for each nominator is $\mathcal {O}(nf)$, where *n* is the number of nodes in the network and *f* is the fraction of nodes required to be identified. The inner loop is about the joint nomination process, related to the number of each nominator’s neighbours. Its computational time is $\mathcal {O}(k_{\max })$, where $k_{\max }$ is the maximum degree of the network. Thus, the computational complexity of the JN algorithm is $\mathcal {O}(nfk_{\max })$. If the required number of identified nodes is fixed, that is, *nf* is constant, then the computational complexity of the JN algorithm will be unrelated to the network scale and the computational complexity decreases to $\mathcal {O}(k_{\max })$, where $k_{\max }$ grows far slower than the network size and the actual node degree is small for the vast majority of the nodes.

Our study is characterized by some limitations. Although the proposed JN strategy performs better than other decentralized strategies in the task of high-degree nodes identification (see Fig. 3), there is still much room to improve, compared to the centralized strategy. Some properties of the networks, such as degree correlation and clustering coefficient, can affect the performance of the JN strategy, which has not been explored in-depth, so more network generated models are needed so that we can create networks with accurately controllable degree correlation and clustering coefficient. The effectiveness of the JN strategy has been testified on static networks but not on dynamic ones, which will be next on our agenda. The proposed JN strategy has not been designed for the networks with community structure, where nodes between different communities are vital for network integrity but do not always have high degrees. Our study uses degree centrality as the metric to measure nodes’ importance, and treats high-degree nodes as vital nodes in the network. Some other metrics, such as betweenness centrality^30^, can also be applied to measure the nodes’ influence. The computational complexity of betweenness centrality is very high, so finding a decentralized strategy without the whole network structure to identifying high-betweenness nodes would be key.

However, this research discovers a more efficient decentralized vital nodes identification strategy. The proposed JN strategy can be used in many cases, such as impeding the spreading of contagious diseases and detecting high-risk accounts in financial networks. This research has also opened a number of avenues for further research. Localized and decentralized vital nodes identification strategies have many advantages over global and centralized strategies, such as lower computational complexity and less information requirement, but suffer from relatively low performance. Some local information can be incorporated, such as neighbours’ degrees, to improve the strategy’s efficiency. The effects of degree correlation and clustering coefficient can be considered to develop a more flexible and effective strategy. The JN strategy can be modified to be applied on temporal networks that are closer to real social contact networks where contagious pathogens spread.

## Methods

### Data description

Numerical simulations are conducted on four undirected-network datasets (one synthetic network and three real networks) and eight directed-network datasets (four synthetic networks and four real networks). Statistical properties of these networks are shown in Table 2.

**Table 2:** Properties of network datasets.

Network	Nodes	Edges	Average degree	Directed	Clustering	Diameter
SYN-BAc	8000	31,980	8	No	0.42	8
G-LastFM	7624	27,806	7.3	No	0.22	15
G-Deezer	28,281	92,752	6.56	No	0.14	21
G-Github	37,700	289,003	15.3	No	0.17	6
SYN-FF-1	8000	24,790	3.1	Yes	0.19	19
SYN-FF-2	8000	40,721	5.1	Yes	0.20	17
SYN-FF-3	8000	96,244	12	Yes	0.23	17
SYN-FF-4	8000	198,713	24.8	Yes	0.29	16
G-Wiki	7115	103,689	14.6	Yes	0.08	7
G-Twitter	81,306	1,768,149	21.7	Yes	0.40	7
G-Email	1005	25,571	25.4	Yes	0.37	7
G-Metabolic	1039	5802	5.6	Yes	0.28	6

**SYN-BAc** ^40^: This synthetic network is generated using the Holme and Kim algorithm^40^. It is based on Barabasi-Albert preferential attachment model^41^, with an extra step that each random edge is followed by a chance of making an edge to one of its neighbours. This model can generate a network with a power-law degree distribution and tunable clustering coefficient. We set $n=8\,000$, $m=4$, and $p=0.9$ respectively.

**G-LastFM** ^42^: This network is a real network dataset from LastFM, an online music service, collected from their public API in March 2020. Each node represents a user. There is an edge between two nodes if they follow each other.

**G-Deezer** ^42^: This is a real network dataset captured from Deezer, an online music service, in March 2020. Nodes are Deezer users from European countries and edges are mutual follower relationships between them.

**G-Github** ^43^: This real network dataset is from Github, collected in June 2019. Each node represents a user who stars at least 10 repositories and edges are mutual follower relationships between them.

**SYNs-Forestfire** ^44^: These directed networks are generated by the Forest Fire model with two parameters: forward-burning probability and backward-burning probability. The Forest Fire model generates networks with power-law degree distributions. We generate four directed networks using this model.

**G-Wiki** ^45^: The wikipedia community held a public vote on order to determine its administrators. A voting network was generated during this process. The network contains voting data from the beginning of Wikipedia until January 2008. Nodes represent wikipedia users, and if one user voted for another, there is a directed link between those two nodes.

**G-Twitter** ^46^: This network is an ego subgraph from Twitter. Twitter users are nodes and there is a directed link from one node to another if that users follows the other.

**G-Email** ^47^: This email network is collected for 112 days at University of Kiel, Germany. Nodes represent email addresses and there is an directed edge between nodes if a user sent more than one email to another.

**G-Metabolic** ^48^: This network reflects the metabolic reactions of the *E. coli* bacteria. Each node is a metabolite, and each directed edge means that there is a reaction where one node is an input and the other is a product.

### Metrics of interest

**Degree distribution**: The degree distribution, $p_k$, provides the probability that a randomly selected node in the network has degree *k*. The degree distribution can capture the full-scale structure of a network.

**Degree centrality**: Degree centrality is the simplest metric to measure a node’s importance: the more links a node has, the greater the importance of the node. Each strategy’s capability of finding hubs is measured by the average degree of the identified nodes.

**Average shortest path length** ^49,50^: The shortest path length between two nodes in a network is defined as the number of edges in the shortest path between these two nodes. The average shortest path length, *L*, of a network is the average of the shortest path lengths over all pairs of nodes, calculated as

2 $$\begin{aligned} \begin{aligned} L = \frac{1}{N(N-1)} \sum _{i,j \in \mathbb {N} ,i \ne j } d_{ij} , \end{aligned} \end{aligned}$$

where *N* is the number of nodes, $\mathbb {N}$ is the set of nodes of the network, and $d_{ij}$ is the shortest path length between node *i* and node *j*.

If there are disconnected components in a network, the average shortest path length will diverge. The average shortest path length of a network is calculated on its largest connected component. The average shortest path length is a strong indicator of a network’s ability to transport pathogens or information. A smaller *L* means a node has a greater probability to become infected, and therefore a high propagation rate.

**Epidemic threshold** ^51^: The spreading rate of a pathogen and the epidemic threshold of a network are two factors that determine how the pathogen propagates in the network. A pathogen’s spreading rate depends on the biological characteristics of the pathogen. A network’s epidemic threshold reflects the capability of the network to resist a pathogen. If the spreading rate of a pathogen exceeds the epidemic threshold of a network, then it will lead to an epidemic on the network, assuming that the spreading rate of a pathogen is constant. Otherwise, the spread of the pathogen dies out. We measure the changes in epidemic threshold in the networks after the removal of the nodes identified by JN and other baseline strategies. Previous work ^51^ indicates that for an SIS (Susceptible-Infected-Susceptible) model, the epidemic threshold, $\tau$, for a network is

3 $$\begin{aligned} \tau = \frac{1}{\lambda _{\max }} , \end{aligned}$$

where $\lambda _{\max }$ is the largest eigenvalue of the network’s adjacency matrix. We use this formula to calculate the epidemic thresholds of networks.

**Infection scale and cumulative infection scale**: Simulating virus spread in a network can be done using Susceptible-Infected-Recovered (SIR) models^52^. The infection scale, *S*(*t*), is the number of infected individuals at any point in time over the total number of individuals, calculated as

4 $$\begin{aligned} S(t) = \frac{n_{I}(t)}{N}, \end{aligned}$$

where $n_{I}(t)$ is the number of infected nodes by time *t*, and *N* is the number of nodes of the network. The cumulative infection scale, $S_{c}(t)$, is the sum of infected individuals plus recovered individuals, over the total number of individuals, calculated as

5 $$\begin{aligned} S_{c}(t) = \frac{n_{I}(t)+n_{R}(t)}{N}, \end{aligned}$$

where $n_{I}(t)$ and $n_{R}(t)$ are the number of infected and recovered nodes by time *t* respectively, and *N* is the number of nodes of a network.
